# A Morpho-Density Approach to Estimating Neural Connectivity

**DOI:** 10.1371/journal.pone.0086526

**Published:** 2014-01-29

**Authors:** Michael P. McAssey, Fetsje Bijma, Bernadetta Tarigan, Jaap van Pelt, Arjen van Ooyen, Mathisca de Gunst

**Affiliations:** 1 Department of Mathematics, VU University, Amsterdam, The Netherlands; 2 Department of Integrative Neurophysiology, VU University, Amsterdam, The Netherlands; Indiana University, United States of America

## Abstract

Neuronal signal integration and information processing in cortical neuronal networks critically depend on the organization of synaptic connectivity. Because of the challenges involved in measuring a large number of neurons, synaptic connectivity is difficult to determine experimentally. Current computational methods for estimating connectivity typically rely on the juxtaposition of experimentally available neurons and applying mathematical techniques to compute estimates of neural connectivity. However, since the number of available neurons is very limited, these connectivity estimates may be subject to large uncertainties. We use a morpho-density field approach applied to a vast ensemble of model-generated neurons. A morpho-density field (MDF) describes the distribution of neural mass in the space around the neural soma. The estimated axonal and dendritic MDFs are derived from 100,000 model neurons that are generated by a stochastic phenomenological model of neurite outgrowth. These MDFs are then used to estimate the connectivity between pairs of neurons as a function of their inter-soma displacement. Compared with other density-field methods, our approach to estimating synaptic connectivity uses fewer restricting assumptions and produces connectivity estimates with a lower standard deviation. An important requirement is that the model-generated neurons reflect accurately the morphology and variation in morphology of the experimental neurons used for optimizing the model parameters. As such, the method remains subject to the uncertainties caused by the limited number of neurons in the experimental data set and by the quality of the model and the assumptions used in creating the MDFs and in calculating estimating connectivity. In summary, MDFs are a powerful tool for visualizing the spatial distribution of axonal and dendritic densities, for estimating the number of potential synapses between neurons with low standard deviation, and for obtaining a greater understanding of the relationship between neural morphology and network connectivity.

## Introduction

The dynamics of neuronal network activity, which underlies all brain functions, depend crucially on the pattern and strengths of synaptic connections between neurons. The formation of synaptic connections between neurons requires physical contact between axonal segments of one neuron and dendritic segments of another neuron. These physical contact sites are potential locations where synapses could be formed; they are called *potential synapses*, since physical contact does not necessarily ensure the formation of a synapse [1]. The occurrence of potential synapses is expected to depend on the geometry of axonal and dendritic arbors, but the relationship between neuronal morphology and synaptic connectivity is not well understood. Investigating how synaptic connectivity depends on neuronal morphology, at the neuron-to-neuron level as well as at the global network level, requires both an accurate representation of the various neuron morphologies and a reliable computational method for translating morphological information into valid estimators of neural connectivity.

In this paper, we use the density field approach in combination with model-generated neurons in order to estimate neural connectivity. Density fields of axonal and dendritic morphologies, which we call *morpho-density fields* (MDFs), describe the statistical distributions of axonal and dendritic mass in the space around the soma. Axonal and dendritic MDFs are also referred to in the literature as fiber densities [2], length densities [3], and statistical representations [4]. We estimate the MDFs using a vast ensemble of model-generated neurons that have been shown to be realistic representations of their biological counterparts based on many statistical properties [5]. These MDFs are then used for estimating synaptic connectivity between neurons. We thereby test the influence of sparsity of morphological data and the impact of assumptions involved in the generation of MDFs. Lastly, we use our MDF approach to generate neural networks and investigate the efficiency of their connectivity patterns.

We build upon the work of Liley and Wright [2] and Kalisman et al. [4]. Liley and Wright [2] developed a method for estimating the expected number of potential synapses between neuron pairs, based on the spatial densities of their axonal and dendritic fibers. These spatial densities are analogous to our MDFs. Their method, which is built upon earlier work by Uttley [6], depends on three limiting assumptions. The first assumption is that the dendritic MDF is spherically symmetric. Second, for the axonal MDF a specific spatial distribution is assumed without clear justification. Finally, it is assumed that the orientations of dendritic and axonal segments are uniformly distributed over the unit sphere. The need for these restricting assumptions lies in the sparsity of experimental data. In order to loosen or drop these assumptions we base the estimated MDFs on a large ensemble of simulated data. Like Kalisman et al. [4] we replace the first assumption by the more realisitic assumption of cylindrical symmetry of the dendritic MDF. The distribution of the dendritic mass in a pyramidal neuron typically shows a cylindrical symmetry around the apical dendrite. We further assume cylindrical symmetry of the axonal MDF for the same reason. We drop the second assumption completely. In [4] the third assumption is dropped, and the actual orientations of the segments are incorporated in the connectivity calculations. Since this yields a considerable computational burden we investigate the influence of this third assumption on the estimated connectivity values.

In both [2] and [4] the methods were applied to limited data of experimentally reconstructed neurons. We demonstrate that this sparsity of experimental data leads to a large variation (i.e., uncertainty) in the connectivity estimates, whereas connectivity values based on MDFs calculated from a vast ensemble of model-generated neurons considerably reduce this variability. To the extent that the MDFs provide a realistic model of the distribution of axonal and dendritic mass about the neuronal soma, and the method for estimating the expected number of potential synapses between two neurons at a given displacement is reliable, the resulting connectivity estimates can provide a better prediction of connectivity in biological neuronal networks.

Biological neurons of any specified type show large variations in their morphology, and the estimation of stable density fields therefore requires large data sets. Sparse experimental data sets will inevitably result in large uncertainties in the density fields and hence in the connectivity estimates. With large data sets of model-generated neurons, however, density fields and connectivity measures can be estimated with much lower variability. Evidently, the model-generated neurons must reflect truly their biological variability. As the parameters of the generating model are optimized on a limited experimental data set, our method remains dependent on the sampling variation in the experimental data, as well as on the quality of the model and the assumptions used in creating density fields and calculating synaptic connectivity.

The paper is organized as follows. In the Methods section we describe the computational method by which estimators of the morpho-density fields can be constructed. Then the different connectivity measures are explained: either based on uniformly-distributed orientations of segments or not. Also a sparse data approach is presented in order to investigate the influence of sparsity. Finally, we describe how a network of neurons can be generated based on the estimated connectivity measures. In the Results section, we show how variation in neuron morphology propagates into uncertainty of connectivity measures. The pyramidal cell morpho-density fields were directly based on the axonal and dendritic arborizations of the model-generated cells, i.e., cylindrical instead of spherical symmetric dendritic fields and no assumed exponentially decreasing axonal field. Moreover, we show that the actual orientations of axonal and dendritic segments do not differ markedly from the uniform distribution assumed in previous studies. Furthermore, we demonstrate that the generated neural networks may be classified as economic small-world networks. The paper concludes with a discussion of the findings.

## Methods

### Generated neurons

We generated 100,000 L2/3 pyramidal neurons from the rat cortex using the NETMORPH software tool [5]. The parameters governing the stochastic growth of the axonal and dendritic arbors for each generated neuron were specified based on an analysis of available experimental neurons, as described in [5]. The estimates of these parameters are consequently subject to sampling variability due to limited experimental data. Nevertheless, Koene et al. [5] demonstrate convincingly that the statistical characteristics of neurons generated by NETMORPH correspond very closely with those of experimentally-reconstructed neurons. In our implementation, NETMORPH simulated 18 days of neuronal development, involving the axon, apical dendrite, and 4 to 8 basal dendrites. The elongation, turning and branching of the growth cones — specialized structures at the tip of growing axons and dendrites — during this process occurred randomly at fixed time increments within the constraints of the specified parameters. Hence, each generated L2/3 pyramidal neuron is posited as a unique and realistic representative from the population of its biological counterparts. Our approach can also be implemented using any other computational model for generating neuronal morphologies.

Pyramidal neurons in rat cortical layers 2 and 3 typically show cylindrical symmetry in the branching patterns. This is due to the orientation of their axonal and dendritic arbors: the axon root segment grows downward from the soma, while the apical dendrite extends upward and the root segments of the basal dendrites have a lateral/downward orientation. Furthermore, as a result of the behavior of the growth cones, the axon and dendrites branch extensively during development to produce large arbors. This cylindrical symmetry is exploited in the sequel.

### Morpho-density fields

Suppose we center the neuron soma at the origin. Let **x** denote an arbitrary point in the space around the soma. Consider all possible axonal morphologies 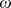 that can develop for a given neuron type, and let 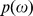 denote the probability density in 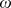. Finally, let 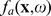 represent the axonal segment mass (measured in length of segment in 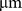) per unit volume for morphology 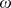 at point **x**. Then the axonal *morpho-density* at **x** is defined as
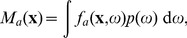
(1)where the integral is taken over all possible axonal morphologies 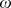. The dendritic morpho-density 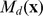 at **x** is defined likewise. The collections 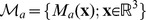 and 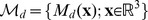 then constitute the axonal and dendritic *morpho-density fields* (MDFs), respectively, for that neuron type.

We constructed estimators for 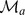 and 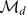 using the large ensemble of 100,000 computer-generated neurons. First of all, we superimposed all generated neurons, such that their spherically-shaped somata are centered at the origin and the 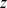-axis is parallel to the apical dendrite. Second, we discretized space into voxels of 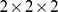 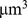. Now, the estimated dendritic MDF at position **x** would equal the average dendritic segment length per 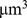 at **x**. However, in a third step we exploited the cylindrical symmetry of the MDFs, as was done in [4]. To this end, we averaged the MDF values over points 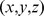 with 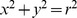, where 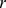 is the horizontal displacement from the vertical 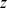-axis. This average morpho-density field was then stored as a function of vertical displacement from the soma (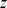) and horizontal displacement from the vertical axis (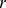). This yielded the estimated dendritic MDF 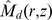, and likewise the estimated axonal MDF 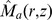. Although these smoothed MDFs were stored in a two-dimensional array, one can easily convert back to the three-dimensional 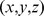 space, taking into account a proper normalization.

### Defining connectivity

**Figure 1:**
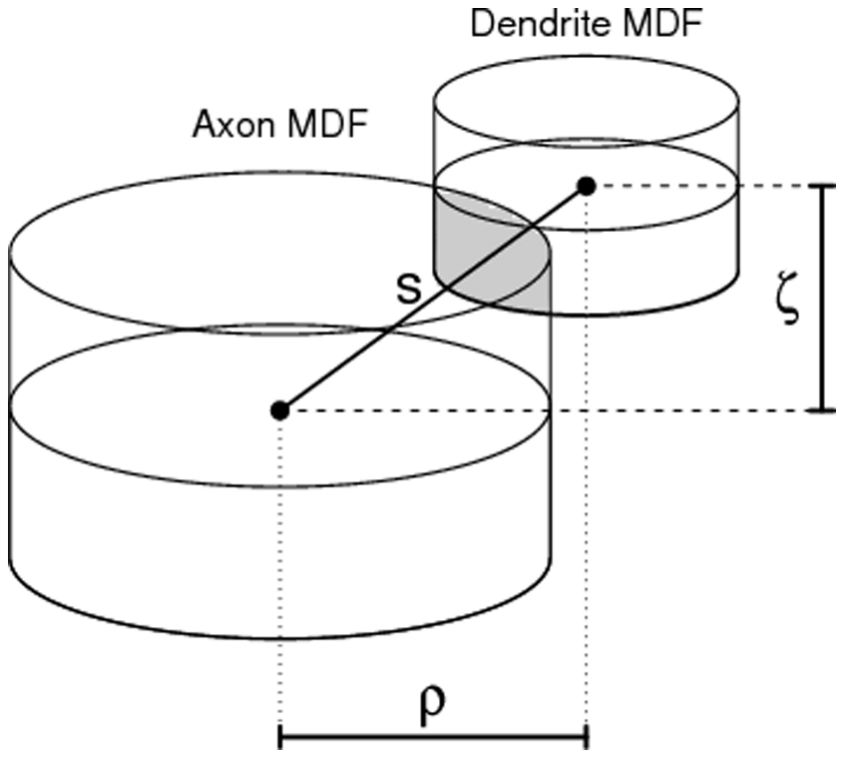
Overlap between the axonal and dendritic morpho-density fields when the soma center of the latter is displaced from that of the former by 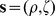.

Connectivity between a pre-synaptic and a post-synaptic neuron is measured by the expected number of potential synapses between the two neurons. A potential synapse is a site where an axonal segment of the pre-synaptic neuron and a dendritic segment of the post-synaptic neuron meet within a certain distance 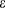. Throughout this study 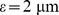. The number of such potential synapses between two neurons of a specific type varies about a certain mean. This mean (or *expectation*) can be computed using MDFs, since the MDFs contain the average segment length density of the neuronal mass. Hence, for any neuron pair we computed 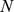, the *expected number of potential synapses*. 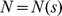 depends on the displacement 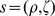 between the somata of the two neurons involved, where 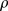 is the displacement in the horizontal 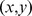-plane and 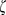 is the displacement in the vertical direction (see Figure 1).

### Connectivity based on uniform orientations

In [2] the expected number of potential synapses is derived, assuming that orientations of both axonal segments and dendritic segments are uniformly distributed over the unit sphere. The formula derived for 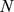 for displacement 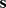 in [2] is
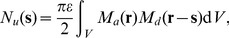
(2)where 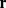 is integrated over 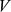, the volume containing the overlap between the two MDFs. The subscript 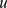 in 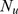 denotes the assumption of uniformly-distributed segment orientations. In [2] 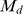 is estimated using Sholl plots of experimentally reconstructed neurons, assuming spherical symmetry of the basal dendrites, and the estimate for 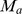 is based on an exponentially decaying distribution that is somewhat ad hoc. In place of 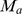 and 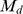, we used our estimated MDFs, 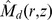 and 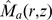, based on the vast representative sample of generated neurons. The estimated number of potential synapses then becomes
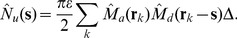
(3)Here the overlap between the two cylindrical volumes containing 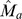 and 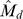 is partitioned into a fine grid of contiguous voxels 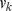 at corresponding locations 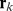, each having volume 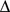. Figure 1 illustrates the two cylindrically symmetric MDFs at displacement 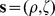, with the overlap region shaded. Using formula (3) we estimated 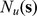 for a range of values of 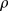 (the horizontal displacement) and 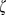 (the vertical displacement).

### Connectivity based on actual orientations

The assumption of uniformly-distributed segment orientations is arguable. Dropping this assumption implies that in the computation of formula (2) the actual orientations of the segments have to be taken into account. This naturally leads to the axonal and dendritic *templates* 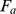 and 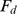 introduced by Kalisman et al [4]. A template 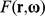 denotes the density of (either axonal or dendritic) segments having orientation 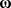 at position 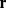. The number of potential synapses then becomes
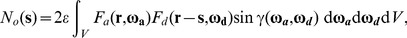
(4)where 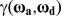 is the angle between orientations 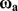 and 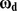. The subscript 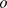 in 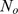 denotes the incorporation of actual segment orientations. In [7] the computation of formula (4) is facilitated by discretizing the range of the orientations into seven principal directions 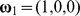, 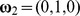, 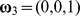, 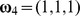, 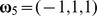, 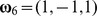, 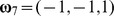. Moreover, in the estimated templates 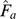 and 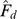 based on experimental L2/3 pyramidal neurons, the cylindrical symmetry of these neurons is exploited. The resulting discretized version of formula (4) is (see [4])
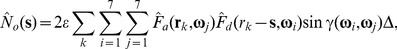
(5)where the sum over 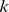 is defined in the same way as in formula (3).

For sparse templates 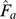 and 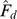, based on only a few neurons, the computation time needed for (5) is comparable to that for (3). However, for non-sparse templates, based on a large number of generated neurons, computing (5) is significantly more time-intensive than computing (3). Therefore, the grid of values of 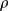 and 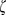 that we used for computing 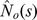 was coarser than that for the computation of 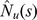.

### Connectivity based on sparse data

To investigate the influence of sparsity of data, we compared our MDF approach to two sparse data approaches. First we computed (5) for simulated data, consisting of 10 to 1000 neurons. For each sample size 20 different data sets were simulated and 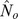 was computed. We anticipated that the 20 estimated MDFs would vary less for larger sample sizes, and, hence, that the variation in the 20 values of 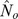 would decrease with sample size. This is quantified in the estimated standard deviation of 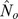 for each sample size. This whole procedure was repeated for different displacements 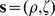.

Second, we applied one of the existing sparse data approaches, the smoothing method presented by Stepanyants and Chklovskii [7]. Their approach to estimating the expected number of potential synapses between neurons uses an estimate of the spatial density of the neurite fibers based on a set of experimental neurons. For each available reconstructed neuron, its axonal and dendritic segment geometries are convolved with a Gaussian kernel to create continuous three-dimensional axonal and dendritic density profiles. The estimated number of potential synapses between a pre-synaptic neuron and a post-synaptic neuron at displacement 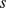 is then computed as
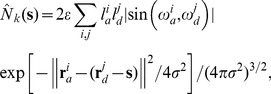
(6)where each individual axonal or dendritic segment is characterized by its position with respect to the soma 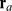 or 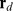, its length 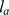 or 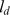, and its orientation 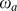 or 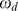, respectively. The subscript 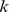 in 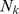 denotes the kernel smoothing approach. This formula involves the two parameters 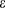 (as in (3) and (5)) and a smoothing parameter 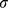 (the standard deviation of the Gaussian kernel). In [7] the range for the latter parameter is given as 10 to 30 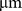. We computed (6) for different displacements and varying values of 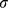.

### Neural networks

Given the estimated number of potential synapses for various displacements, it is possible to generate a random directed weighted neural network to represent L2/3 pyramidal neurons in the rat cortex. The vertices represent the neurons, and each directed edge from one vertex to another represents a potential synaptic connection from the pre-synaptic neuron to the post-synaptic neuron. The weight of the connection between any neuron pair represents the strength of the connection and is based on the estimated number of potential synapses. We randomly generated locations (the vertices) for the somata of simulated pyramidal neurons within a cylinder with no two vertices closer than 20 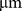. For each pair 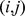 of vertices we computed 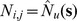 with 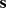 the directed displacement between the two vertices.

Once a neural network has been generated, the efficiency of its connectivity pattern can be investigated. In biological neural networks, strong inter-connectivity among neighboring neurons may enhance local computational efficiency, and short paths between local clusters may enhance the transmission of information throughout the network [8]–[11]. For binary graphs the *small-world coefficient* is typically used for assessing the efficiency of the network [11], [12]. This coefficient depends on shortest path lengths and cluster coefficients. However, since the definition for the cluster coefficient of weighted graphs has not yet been settled, the small-world coefficient is not directly applicable for weighted directed graphs. Latora and Marchiori have proposed a proper alternative for weighted graphs in their efficiency measures [13]. In their approach a weighted graph is given by its adjacency matrix 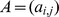 and its weight matrix 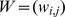. The adjacency value 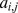 equals 1 if the connection from node 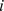 to 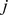 exists, and equals 0 otherwise. The weights 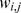 are given for all connections, including the connections that do not exist, i.e. those with 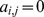. The *efficiency* of a weighted graph 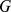 consisting of 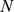 vertices is then defined as
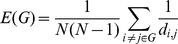
(7)where 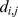 is the shortest path from 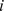 to 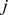. The length of a path is defined as the sum of the reciprocals of the weights of its edges. Small weights correspond to long/weak connections, whereas large weights represent short/strong connections. The *global efficiency* of a graph is
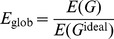
(8)where 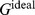 is the weighted graph with all 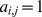. The *local efficiency* is
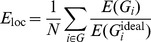
(9)where 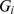 is the weighted subgraph consisting of neighbours of vertex 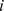. Both these efficiency values are between 0 and 1. For binary networks, global efficiency is closely related to the average shortest path length, while the local efficiency value expresses the local connectivity, like the cluster coefficient for binary graphs. The *cost* of a weighted network expresses the weighted number of realized connections and is defined as
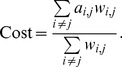
(10)An *economic small-world* network has high global and local efficiency, while the cost of such a network is low [13]. Such networks can efficiently process information at low infrastructural cost.

In order to apply these efficiency measures to our generated neural networks, we needed to define both an adjacency matrix and a weight matrix for each generated network. The adjacency value 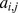, representing the existence of a connection from 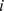 to 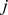, was generated in the following way: we randomly generated each 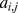 as the outcome of a Bernoulli trial with success probabilty equal to 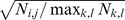. The elements of the weight matrix were defined by 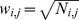. Using this combination of 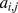 and 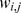, we ensure that the expectation of 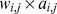 is linear in 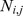. In other words, the realized weighted connection strength between two neurons in the generated network scales linearly with the expected number of potential synapses between them.

## Results

### Estimated morpho-density fields

**Figure 2:**
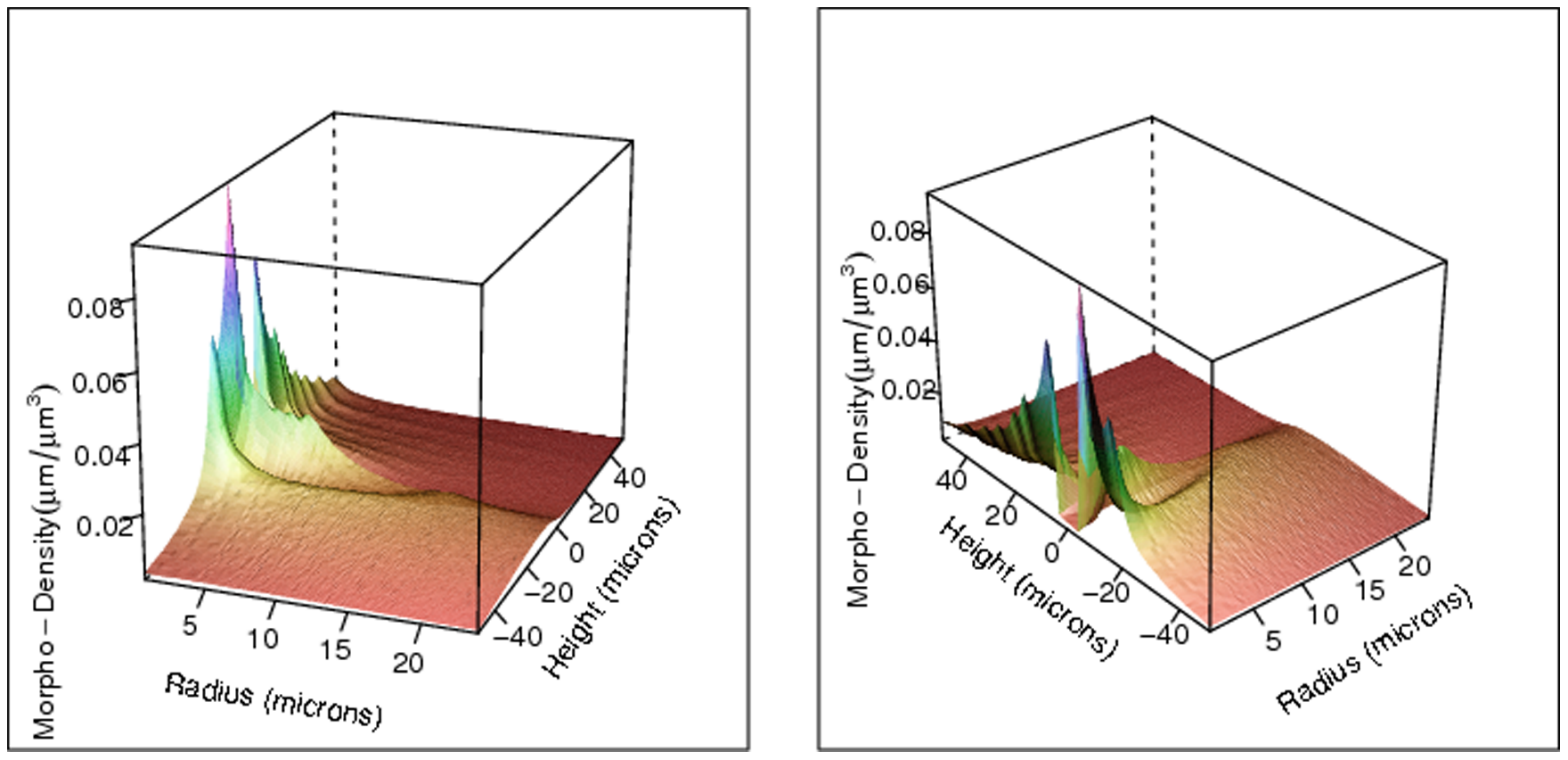
The estimated morpho-density for the dendritic arbors near the neural soma with respect to the radius 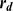 and the height 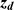 (both in microns), with 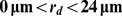 and 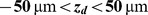, from two perspectives. Note the different scales for the radius and height axes.

Figure 2 displays the value of the estimated MDF 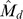 for the dendritic arbors near the neural soma as a function of the radial distance 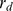 and height 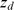, based on an ensemble of 100,000 L2/3 pyramidal NETMORPH-generated neurons. The left and right panel show the same estimated MDF from two different perspectives. Note that this dendritic morpho-density estimate has a peak at about 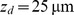 for small 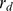, corresponding to the position of the apical dendrite above the soma before it branches off in all directions. The morpho-density is low at the soma (around 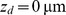), then has a taller peak at about 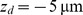, corresponding to the extension of the basal dendrites downward and away from the soma. Basal dendrites generally spread out as they extend from the soma, accounting for the ridge in the morpho-density for 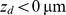 and 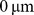 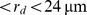. As we move away from the soma, the morpho-density decreases to very small values, due to the dispersion of the dendrite arborization over the surrounding volume.

**Figure 3:**
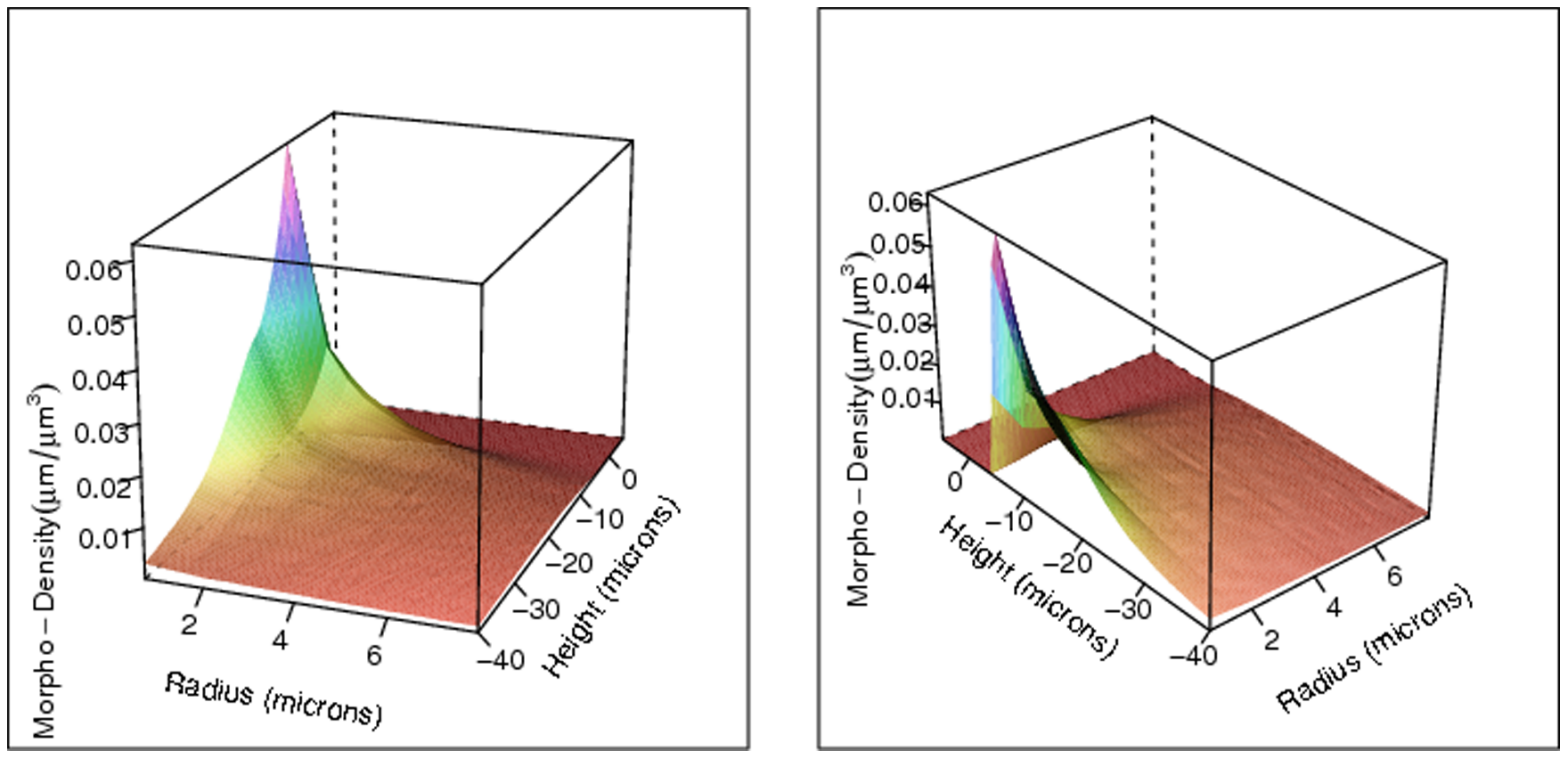
The estimated morpho-density for the axonal arbors near the neural soma with respect to the radius 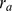 and the height 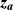 (both in microns), with 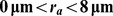 and 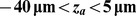, from two perspectives. Note the different scales for the radius and height axes.

Similarly, Figure 3 displays the value of the estimated MDF 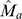 for the axonal arbors near the neural soma as a function of the radial distance 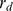 and height 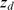, based on the same ensemble, from two different perspectives. The axonal morpho-density has a single peak just below the soma, from which the axon emerges before branching away. As we move away from the soma, the morpho-density decreases to very small values, due to the extensive dispersion of the axon over the surrounding volume.

**Figure 4:**
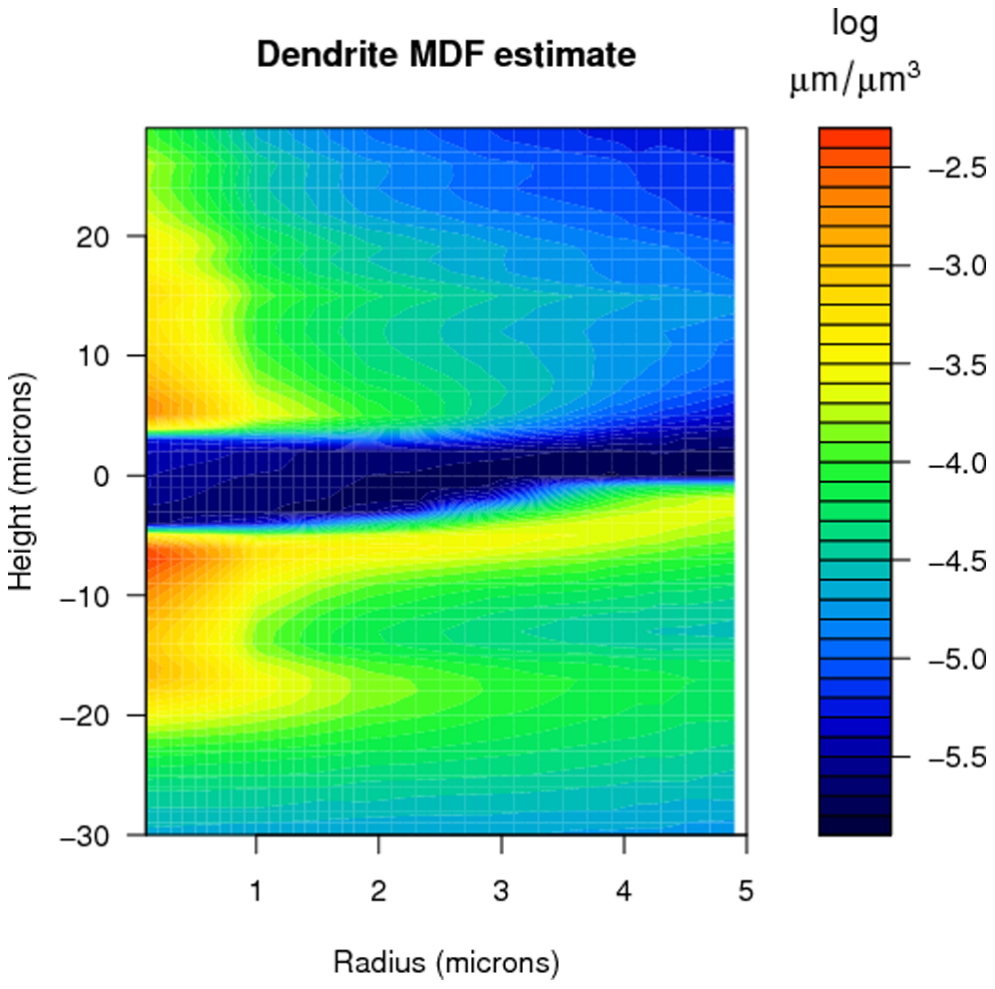
Heat map of the logarithm of the estimated morpho-density in Figures 2, for small values of the radius 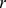 and height 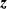.

**Figure 5:**
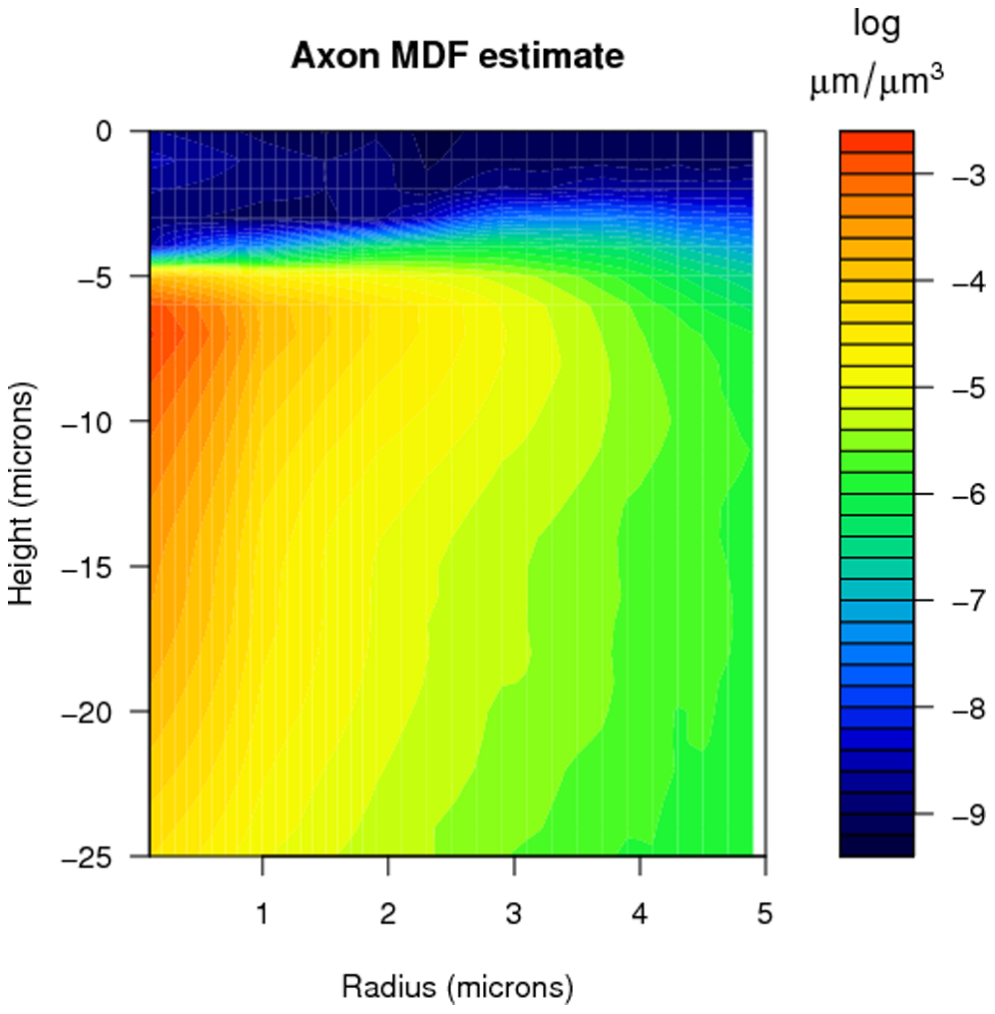
Heat map of the logarithm of the estimated morpho-density in Figures 3, for small values of the radius 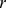 and height 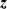.

The heatmaps in Figures 4 and 5 give yet another visualization of the morpho-densities near the soma. Figures 2, 3, 4 and 5 demonstrate an additional advantage of using a large generated data set instead of a sparse data set: given ideal simulated neurons, the morpho-densities of biological neurons can be estimated and visualized for a fine resolution of radius and height values, 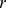 and 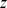. This resolution would increase as the voxel size decreases.

### Estimated connectivities

**Figure 6:**
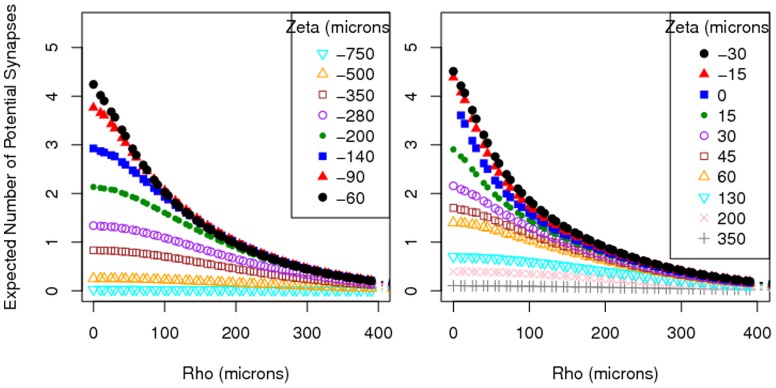
Estimated number of potential synapses 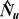 between the axonal arbor of one L2/3 pyramidal neuron and the dendritic arbor of another when the latter is horizontally displaced by 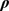 microns and vertically displaced by 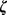 microns, based on formula (3), assuming uniformly distributed segment orientations.

Figure 6 shows 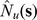, the estimated number of potential synapses (3) assuming uniformly-distributed segment orientations, for various values of 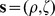, based on MDF estimates made from 100,000 simulated neurons. The figure demonstrates the continuity of the function 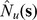 over its domain. This suggests that 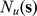 can be reasonably estimated for any 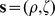 by interpolating among the stored grid of previously-computed values without having to resort to (3) in future instances. These values for 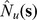 are consistent with the corresponding estimates presented in earlier studies of connectivity among L2/3 pyramidal neurons (see [1]).

**Figure 7:**
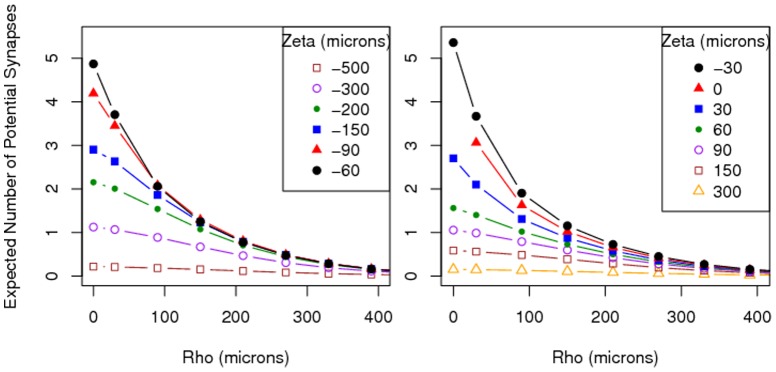
Estimated number of potential synapses 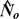 between the axonal arbor of one L2/3 pyramidal neuron and the dendritic arbor of another when the latter is horizontally displaced by 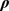 microns and vertically displaced by 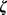 microns, based on formula (5), using actual segment orientations.

Figure 7 shows 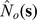, the estimated number of potential synapses (5) using the actual segment orientations (rounded to the nearest principal direction), for various values of 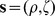, based on 100,000 generated neurons. Since the computation of 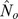 is significantly more time-intensive than the computation of 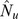, the grid for 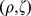 taken in Figure 7 is coarser than that in Figure 6. Nevertheless, the shapes of the lines for the different 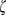 values look similar to those in Figure 6.

**Figure 8:**
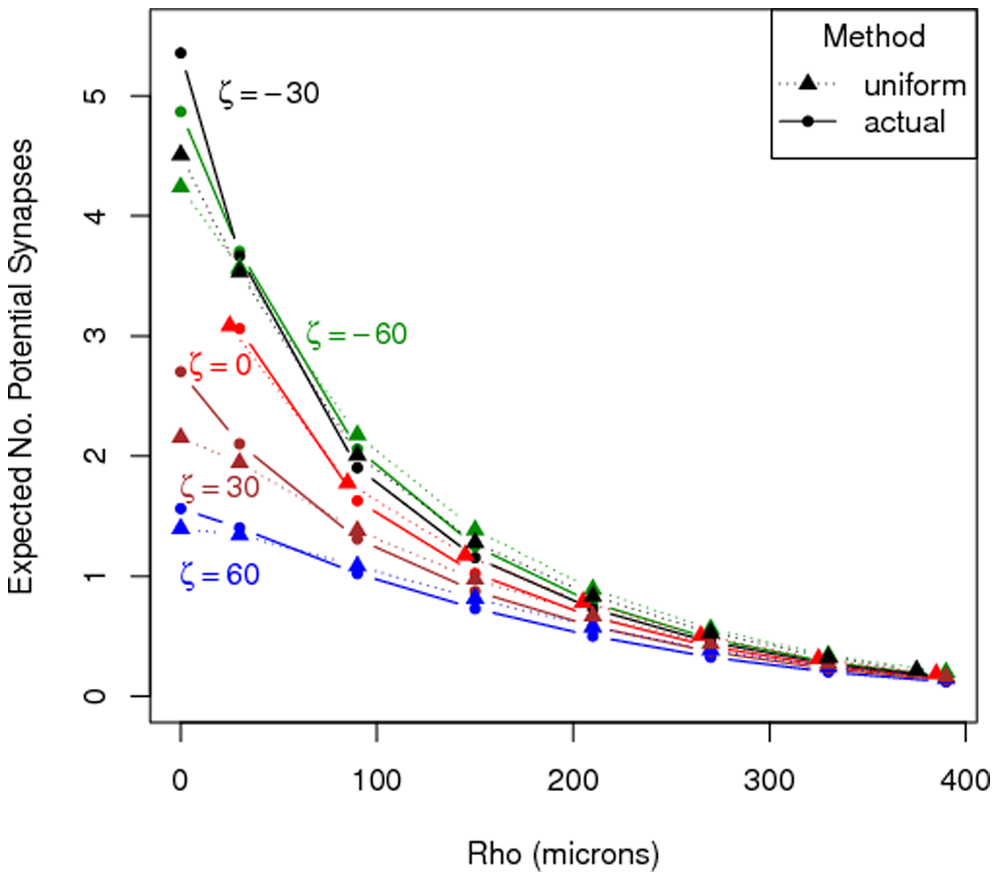
A selection of the values for 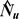 from Figure 6 (▴), and 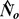 from Figure 7 (•).

In Figure 8 a comparison of the two methods is made for five values of 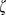. It shows both 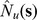 based on (3) and 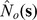 based on (5) for a selection of 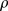 and 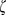 values. For 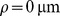 and small 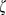 the estimated connectivity values 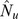 assuming uniformly-distributed segment orientations are lower than the estimated connectivity values 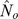 based on actual segment orientations. A displacement with 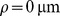 means that there is only a vertical shift between the somata of the two neurons. Hence, the apical dendrites are partly overlaid. The orientation of the apical dendrite is far from uniformly distributed, since it is predominantly vertical. This causes the difference between the two methods for 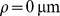. Figure 8 shows that this difference decreases when vertical displacement increases, i.e. when 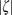 increases. For larger 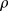 values differences are hardly noticeable and insignificant (see Figure 8).

**Figure 9:**
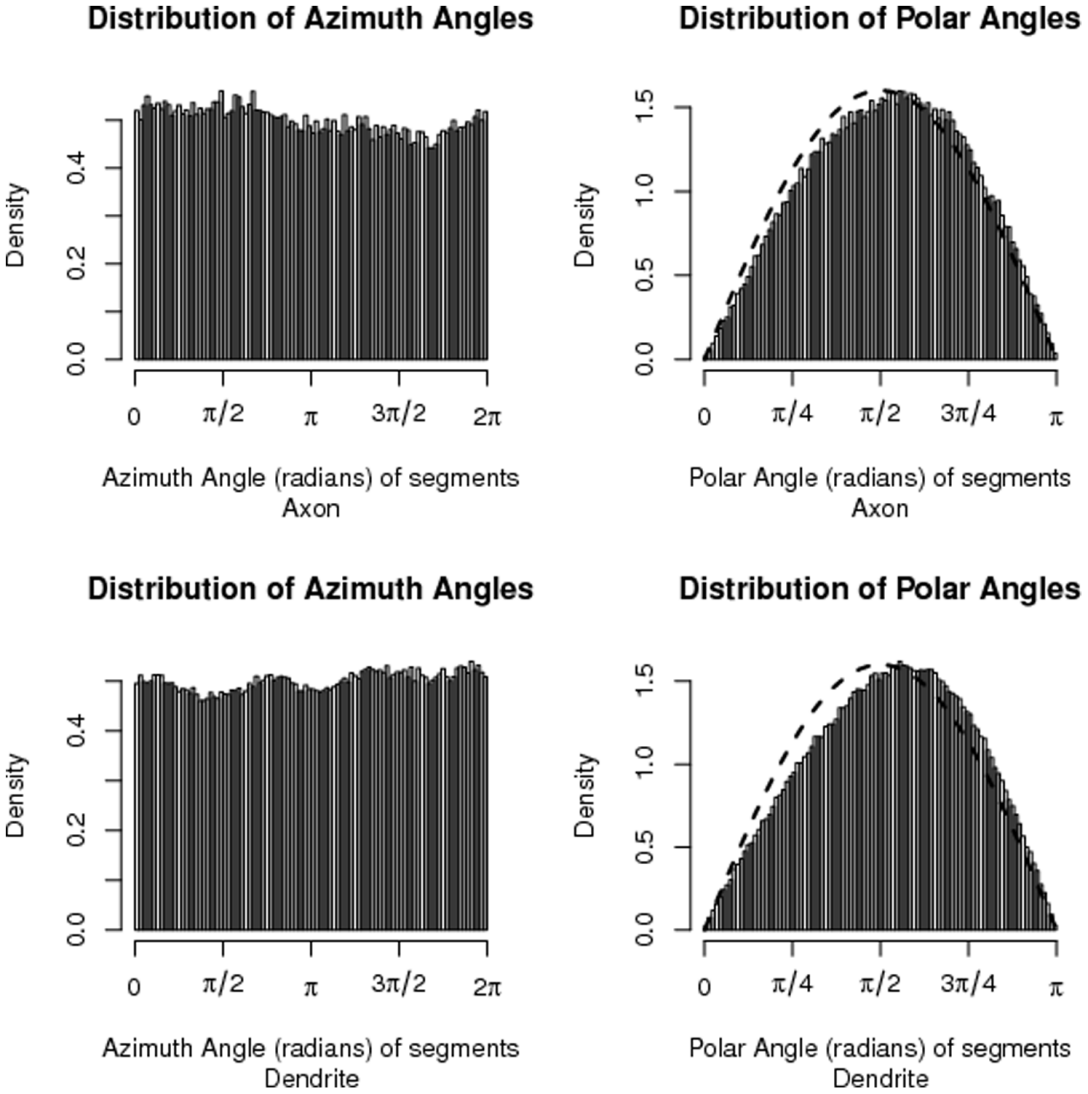
Frequency distribution of the azimuthal and polar angles of the orientation of axonal and dendritic segments among 100 NETMORPH-generated neurons. A superimposed sine curve shows the slight skewness of the polar angle distribution.

From Figure 8 it appears that, apart from the apical dendrite, the orientations of the segments seem not to differ from uniform over the unit sphere substantially. To investigate this further we generated 100 L2/3 pyramidal neurons using NETMORPH. For each neuron we took an inventory of its axonal and dendritic segments and their respective orientations, that is, their azimuthal and polar angles in radians. Histograms of the azimuthal and polar angles are shown in Figure 9 for dendritic and axonal segments separately. When the orientation is uniformly-distributed over the unit sphere, the distribution of the azimuthal angle is uniform between 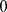 and 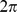 radians and the polar angle has a sinusoidal distribution between 0 and 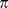 radians. It appears from Figure 9 that the azimuthal angles comply with this assumption, whereas the distribution of the polar angles is slightly skewed in both dendritic and axonal segments (compare with the superimposed sine curves). This indicates that the orientations are directed somewhat more downwards than one would expect under a perfect uniform distribution of the orientations. Such a small skewness may be expected as the initial dendritic root segments at the start of neuronal development have a lateral/downward orientation, while the axonal root segment is fully downward oriented. Nevertheless, the deviation is very small, and does not play a visible role in the estimated connectivities.

### Results based on sparse data

**Figure 10:**
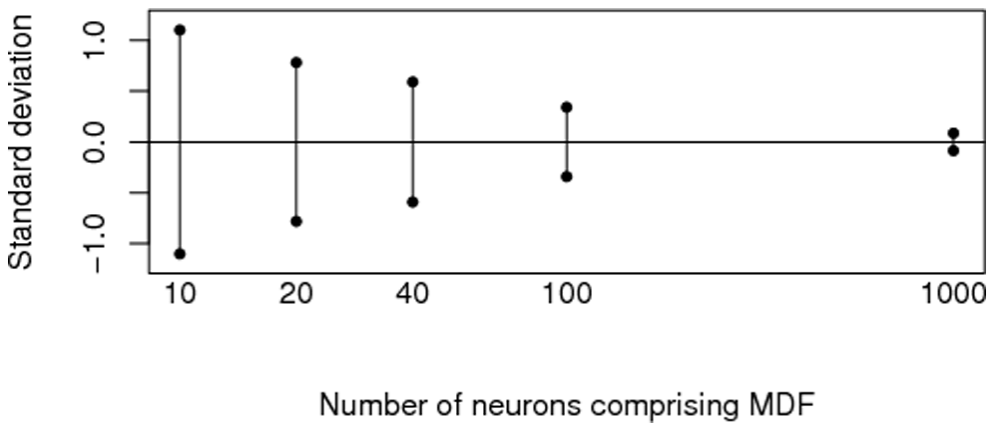
Estimated standard deviations for 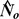 based on sparse data of varying sample size for displacement 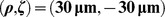.

In the first sparse data approach we investigated the influence of sparsity on the variability (i.e., standard deviation) of the estimated number of potential synapses 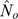. To this extent, we computed 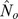 for 20 MDF pairs, with each pair based on a different sample of model-generated neurons, and estimated its standard deviation from the variation in the 20 obtained 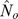 values. Using NETMORPH, 20 different MDF pairs were generated, each comprised from a sample of 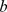 virtual L2/3 pyramidal neurons, with 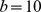, 20, 40, 100 and 1000, respectively. For each sample size 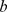 we created the estimated axonal and dendritic MDFs, and computed 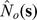 for 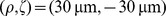. This yielded 20 estimates 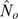 for each sample size 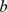. Since the variation in the estimated MDFs propagates to the 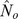 values, the standard deviation of the so-obtained 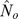 values will drop with increasing sample size. In Figure 10 these estimated standard deviations are shown as a function of sample size. The estimated standard deviation drops significantly between small sample size (10 neurons) and larger sample size (1000 neurons). This figure shows that connectivity measures based on sparse data suffer from the large variability in such data, which is reflected in the large standard deviation for small sample sizes. The MDF approach is based on a sample size of 100,000 model-generated neurons, which clearly has a much smaller standard deviation (by extrapolation of the figure). Consequently, a confidence interval for 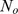 based on a very large sample will be quite narrow, while one based on a small sample of experimental neurons must of necessity be rather large.

To investigate the dependence of the standard deviation on the displacement, 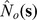 was computed for 14 different values of 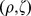, using a sample size of 10 neurons. Table 1 displays for each displacement the mean, standard deviation, minimum and maximum values of these connectivity values. It appears that the variability in the estimated connectivity is large for all displacements. Reported standard deviations are about 20 to 30% of the mean, irrespective of the displacement.

**Table 1:** Variability of estimates using (5).

(*ρ* µm, *ζ* µm)	mean	std.dev.	min	max
(0, −30)	4.161	1.325	2.388	5.887
(30, −30)	3.110	0.921	1.883	4.688
(60, −30)	2.173	0.520	1.325	2.830
(90, −30)	1.626	0.331	1.075	1.991
(120, −30)	1.247	0.230	0.918	1.551
(30,0)	2.549	0.801	1.565	3.933
(60,0)	1.832	0.545	1.045	2.754
(90,0)	1.413	0.372	0.872	1.979
(120,0)	1.094	0.267	0.693	1.433
(0,30)	1.884	0.754	1.016	3.044
(30,30)	1.754	0.698	0.956	3.013
(60,30)	1.455	0.53	0.797	2.384
(90,30)	1.206	0.393	0.758	1.983
(120,30)	0.946	0.264	0.609	1.409

Variability in the estimation of the expected number of potential synapses at 14 different displacements 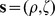 using the formula (5) based on axonal and dendritic templates created from 9 different sets consisting of 10 generated L2/3 pyramidal neurons each. Values given are mean, standard deviation, minimum and maximum of 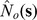 for indicated displacement 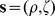.

In the second sparse data approach we applied the smoothing method (6). We generated 10 NETMORPH L2/3 pyramidal neurons and stored the positions, lengths and orientations for all neurite segments comprising each arbor. As in [7], we combined in the computation of 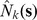 the axonal template and dendritic template of one neuron. This yielded 10 combinations. For two fixed displacements 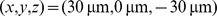 and 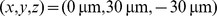 we computed 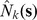 for each of the 10 combinations for 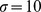, 20 and 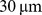, using (6). Note that for both displacements we have 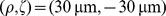, i.e., the displacements are equivalent with respect to the cylindrical symmetry. The results for the two equivalent displacements did not differ significantly. Therefore, we show in Table 2 the mean, standard deviation, minimum, maximum values of 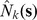 for each 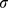, pooled over the two equivalent displacements. Although in [7] it is stated that 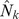-values do not depend strongly on the smoothing parameter 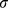, we find that the 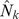-value drops significantly with increasing 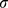. Moreover, the variability of 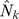 is high, given that the estimated standard deviation is larger than the mean. Thus connectivity estimates based on sparse data using the smoothing method have large sampling variability.

**Table 2:** Variability of estimates using (6).

*σ* µm	mean	std.dev.	min	max
10	3.121	3.656	0.134	13.321
20	2.575	3.049	0.168	11.382
30	2.143	2.483	0.155	9.356

Results for the sparse data approach, using formula (6). Given numbers are the mean, standard deviation, minimum and maximum of 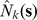 for each indicated value of *σ*. For each line 20 values for 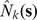 are generated, based on 10 axon-dendrite template combinations and on two symmetrically equivalent displacements, 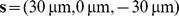 and 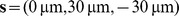.

### Economic small-world property

Three neural networks were randomly generated using the 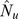 values, as described in the Methods section. Each generated neural network consisted of 2000 neurons. These 2000 vertices were distributed uniformly in a cylinder. Three different shapes were used for the cylinder: a tall pipe, a flat disc and an intermediate cylinder. These three were chosen in order to check whether the economic small world property of a neural network depended on the shape of the volume containing it. The three cylinders had the same volume, such that the density of each the cylinder was 75,000 neurons/mm^3^ (comparable to layers 2 and 3 of the rat cortex [1]). We maintained a minimum distance of 20 microns between soma pairs since the soma radius is about 10 microns.

Table 3 shows the values of the global and local efficiency for the three networks. Both local and global efficiency values are very high (>85%) for all three shapes of the cylinder, reflecting a highly efficient network in each case, and demonstrating that this efficiency is robust to the shape of the cylinder. The cost value, shown in the most right column, is extremely low for all three shapes, and decreases with decreasing height of the cylinder. This is due to the fact that the 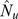 values decrease relatively more rapidly for increasing 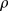 (horizontal displacement) than for increasing 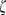 (vertical displacement), as shown in Figure 6. In the bottom line of Table 3 the displacements are mainly horizontal, leading to small values of 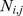, and hence, a sparse adjacency matrix which results in a low cost value.

**Table 3:** Local and global efficiency.

radius (µm)	height (µm)	global efficiency	local efficiency	cost
130	500	0.8685	0.8663	0.005500
200	212	0.8583	0.8563	0.005600
500	34	0.9175	0.8930	0.000022

Local and global efficiency values of the generated network consisting of 2000 neurons, placed in a cylinder of the indicated radius and height. The right-most column shows the cost values of the networks.

The reported efficiency values are comparable to values reported for the human brain network, which range up to 85% [14]. Reported efficiency values for transportation networks are around 70%, with a cost value similar to the cost values in Table 3 [15]. Such networks can be classified as economic small-world networks, which are characterized by high global and local efficiency combined with a low cost. We conclude here that for each cylinder the generated neural network may thus be classified as an economic small-world network. Small-world topology supports efficient communication between neurons at both the local and global levels while minimizing the demand for resources [8]–[11].

## Discussion

In this paper we have presented a morpho-density field approach based on model-generated neurons to estimating neural connectivity. Morpho-density fields (MDFs) of axonal and dendritic morphologies describe the statistical distributions of axonal and dendritic mass in the space around the soma. The MDFs are extremely useful for visualization of the density of neurite segments for any specified neuron type. The vivid detail in the MDFs, as demonstrated in Figures 2, 3, 4 and 5, is made possible by the ability to generate a large ensemble of simulated neurons using software such as NETMORPH. By using such tools to estimate axonal and dendritic MDFs, it will be possible to create detailed characteristic morphological profiles of different types of neurons to an extent which cannot be accomplished using small samples of experimentally reconstructed neurons. These MDF profiles can subsequently form the basis for the investigation of neural networks and their synaptic connectivity.

In [1], [7], [16], [17] other approaches to estimating connectivity have been presented. All these studies represent the spatial densities of the neuronal fibers through extrapolations from a small set of experimental neurons, and use these spatial densities to calculate connectivity measures. Braitenberg and Schüz [16] projected the dendritic arbors onto a plane perpendicular to the axonal direction, and made the probability of a connection proportional to the dendritic density on the projected plane. Hellwig [1] counted potential synapses occurring between pairs of experimentally reconstructed axonal and dendritic arborizations digitally juxtaposed over a range of distances, and used the averages over these pairs to compute connection probabilities. Amirikian [17] used synaptic density fields based on observed potential synapses occurring on available two-dimensional drawings of neurons to estimate the number of synaptic contacts for different displacements. Stepanyants and Chklovskii [7] convolved the locations of neurite segments of reconstructed neurons with a Gaussian kernel in order to accomodate the variability in arbor geometries and measurement imprecision due to small sample sizes. Of all these approaches, the latter one is closest to the MDF approach, since it defines neural density in the space, which has a similar interpretation to that of the MDF. Nevertheless, we have shown that the MDF approach, using generated neurons from a parametric outgrowth model, results in estimates of connectivity which have a much smaller variability than estimates obtained using the kernel-smoothing method in [7].

In contrast to these approaches described above, our MDF approach is not based on a limited data set of neuron morphologies, and does not rely on several restricting assumptions characteristic of other approaches: spherical symmetry of dendritic fields [2], a radial exponentially-decreasing axonal density function [2], a uniform distribution of dendritic and axonal segment orientations [2], or a smoothing method to create density profiles [1], [7], [16], [17]. We have shown here that sparsity of data produces connectivity estimates with high sampling variability. Using the morpho-density fields, this variability is greatly reduced.

Our obtained estimates 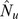 and 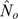 fall within the range of corresponding estimates reported in [1] for L2/3 pyramidal neurons at equivalent displacements. However, rather than depending on a pool of relatively few representatives of a neuron's morphology and averaging the numbers of potential synapses between neuronal pairs over just a few symmetrically-equivalent displacements, we rely on a vast number of morphologies. Hence, the connectivity estimates based on the MDF approach have a very small standard deviation, as illustrated in Figure 10.

Nevertheless, it should be acknowledged that the sparsity of experimental data affects the MDF approach as well. That is because the estimates of the NETMORPH parameters are derived from the morphological characteristics of a single set of experimental neurons. Because of the lack of large datasets of reconstructed neurons, we could not investigate how the connectivity estimates would change if another sample of neurons was used. Another sample, or an extension of the current sample, could result in different experimental distributions, different NETMORPH parameters and consequently also different connectivity estimates. Whether these differences would be non-negligible requires further investigation. The availability of experimental data on neuronal morphologies will increase over time, and methods for deriving estimates for these parameters will keep improving, so that the generated neurons which form the basis of our estimators of the MDFs will become even more realistic representations of biological neurons. Meanwhile we have demonstrated convincingly that, given a fixed set of experimental neurons, we obtain connectivity estimates with a much smaller standard deviation using the MDF approach (Figure 10, Table 1) based on that set than we would if we used a sparse data method (such as the Gaussian convolution approach in (6), Table 2) on the same set.

The MDFs do not carry spatial correlation information. It is certainly the case that, in a single instance of a neuron, the presence of a neurite segment at one location increases the likelihood of segments being found concurrently at neighboring locations. In order to take this spatial correlation into account, one would need to store the full arbor geometry densities 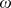 jointly, instead of storing the densities of the locations marginally, as in (1). This poses a condition on computational resources that currently cannot be met.

We have further shown that the assumption of uniformly-distributed segment orientations is not violated significantly in the ensemble of generated neurons. The histograms in Figure 9 agree with a uniform distribution on the unit sphere to a large extent. The small deviation from the sinusoidal distribution of the polar angle does not lead to a systematic under- or over-estimation of the connectivity, apart from the small deviations for 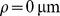 (see Figure 8). Hence, once may avoid the computational burden of the templates in (5) and use (3) instead.

The generated networks based on the estimated connectivity values appear to be economic small-world networks in terms of global efficiency, local efficiency and cost values. However, it is still an open question whether these measures are optimal for quantifying efficiency of weighted networks. Different approaches to answering this question are currently being investigated (see the review in [18] and references therein). In any case, we have demonstrated that the ability to estimate connectivity among neurons in this manner provides a simple tool for investigating connectivity properties of neural networks.

The present study has shown how the uncertainty in the expected number of potential synapses between two neurons depends on the size of the data set, as visualized in Figure 10 for L2/3 neurons at a given distance. As these uncertainties find their origin in the variability between neuronal morphologies, it is expected that the connectivities, measured between actual neurons in experimental studies, will show at least similar variability. As such, the presented MDF approach may be helpful in estimating the number of neuron pairs required if a connectivity estimate with a given uncertainty (standard deviation) is desired.

In summary, our morpho-density approach to estimating neuronal connectivity incorporates the characteristics of neuronal growth and network formation without being directly dependent on small data sets. We have shown that the so-obtained estimated connectivity values have a much lower standard deviation than connectivity values based on sparse data. Moreover, this approach is not restricted to L2/3 pyramidal neurons, but can be applied to any type of neuron, and combinations of different types. Therefore, we anticipate that our approach may serve as an important tool for analyzing the shapes of neuronal morphologies as well as the generation and study of synaptic connectivity of neural networks.
